# The Function of Fish Cytokines

**DOI:** 10.3390/biology5020023

**Published:** 2016-05-24

**Authors:** Jun Zou, Christopher J. Secombes

**Affiliations:** Scottish Fish Immunology Research Centre, University of Aberdeen, Zoology Building, Tillydrone Avenue, Aberdeen AB24 2TZ, UK; j.j.zou@abdn.ac.uk

**Keywords:** cytokines, bioactivity, recombinant proteins, loss- and gain-of-function, interleukins, interferons, colony stimulating factors, tumour necrosis factors, transforming growth factor-β

## Abstract

What is known about the biological activity of fish cytokines is reviewed. Most of the functional studies performed to date have been in teleost fish, and have focused on the induced effects of cytokine recombinant proteins, or have used loss- and gain-of-function experiments in zebrafish. Such studies begin to tell us about the role of these molecules in the regulation of fish immune responses and whether they are similar or divergent to the well-characterised functions of mammalian cytokines. This knowledge will aid our ability to determine and modulate the pathways leading to protective immunity, to improve fish health in aquaculture.

## 1. Introduction

It is clear that all of the major cytokine families exist in fish, as outlined in many previous reviews that have described the genes present and when/where they are expressed at the transcript level [1,2,3,4]. In many cases, multiple copies (paralogues) of particular genes occur (particularly common in the teleost fish) and independent expansion of gene families in different lineages has taken place from putative common ancestral genes. For example, the classical pro-inflammatory cytokines IL-1β, TNF-α and IL-6 are present, with multiple paralogues in most species. Similarly, cytokines associated with adaptive immunity are present, including IL-2, IFN-γ, IL-4/13 (with homology to IL-4 and IL-13), IL-10, IL-17A/F (with homology to IL-17A and IL-17F), IL-21, IL-22 and TGF-β1 (amongst others), also with multiple paralogues. Hence it seems likely that many aspects of the regulation of immune responses in vertebrates are ancient and relatively well conserved, assuming conservation of function based on our understanding of the biological effects of primarily mammalian molecules. Since few antibodies exist to fish cytokines, there is virtually no data on protein expression, with a couple of exceptions (e.g., IFN and TNF-α). Thus most of the functional studies on fish cytokines have either investigated the bioactivities of cytokine recombinant proteins, produced using primarily prokaryotic (e.g., *Escherichia* *coli*) expression systems and on a few occasions in eukaryotic cell lines (fish and mammalian), or they have used loss- and gain-of-function experiments in model species, such as zebrafish. Such studies, primarily in teleost fish, begin to tell us about the role of these molecules in early vertebrates and whether they are similar or divergent between paralogues and to the well-characterised functions of mammalian cytokines. What is known about cytokine bioactivity in fish will be the focus of this review. The cytokines have been grouped by their molecular structure since this dictates the receptors used and the down-stream signalling pathways that are activated. However, the “open face β sandwich” cytokines (chemokines) are excluded since these are described elsewhere in this special issue [5]. It will become apparent that we now know a lot about the function of many fish cytokines, albeit in relatively few fish species at this point in time.

## 2. β-Trefoil Cytokines

The beta trefoil cytokine family, also termed the IL-1 family, consists of 11 members (IL-1F1-11) in mammals and are central players in regulating inflammation [6,7]. IL-1F1-4 are also referred to as IL-1α, IL-1β, IL-1 receptor antagonist (IL-1Ra) and IL-18, with the other members (IL-1F5-11) assigned as IL-33 (IL-1F11), IL-36α (IL-1F6), IL-36β (IL-1F8), IL-36γ (IL1F9), IL-36 receptor antagonist (IL-36Ra, IL-1F5), IL-37 (IL-1F7) and IL-38 (IL-1F10). Functionally, the IL-1 family cytokines can be classified into two groups, either promoting (IL-1α, IL-1β, IL-18, IL-33 and IL-36α, β, and γ) or suppressing (IL-1Ra, IL-36Ra, IL-37 and IL-38) inflammation.

IL-1β was the first interleukin characterised in bony and cartilaginous fish [8,9,10,11,12,13,14,15]. Later, two other members of the IL-1 family were discovered in fish, namely IL-18 and a teleost specific group termed novel IL-1 family member (nIL-1Fm) [16,17,18]. Extensive analysis of fish genomes has confirmed that fish lack orthologues to other mammalian IL-1 family members [1]. Teleost fish IL-1β genes exist as multiple copies that have recently been classified into two groups (type I and II) based on the genomic organisation and gene synteny [11,15,19,20,21,22,23,24,25]. Type I IL-1β genes have a seven exon/six intron organisation, which is conserved among all the jawed vertebrates. In contrast, teleost type II IL-1β genes appear to be a lineage specific group containing six or five exons and are believed to have arisen from a common ancestral gene that gave rise to the type I IL-1β genes. However, the phylogenetical tree analysis revealed that the two teleost IL-1β groups are closely related and form a separate clade relative to IL-1β genes from other vertebrates. Not all fish species possess both groups, for example, type I but not type II IL-1βs are found in cypriniformes [20]. A single copy gene of the IL-18 orthologue has been reported in a limited number of fish species but its immune functions have yet to be determined [16,18]. The nIL-1Fms are a teleost specific group of the IL-1 family and have a similar function to mammalian IL-1Ra in antagonising IL-1β activities [17,22,24]. However, they are not 1:1 orthologues of mammalian IL-1R antagonists.

### 2.1. IL-1β

IL-1β is produced by a wide range of cell types after activation of host pattern recognition receptors (PRRs) by pathogen associated molecular patterns (PAMPs) or danger associated molecular patterns (DAMPs) [26,27,28,29]. The IL-1β is synthesised as a precursor lacking a signal peptide and is required to be processed to release active protein [30]. The IL-1β precursor can be cleaved either intracellularly or extracellularly [31]. In humans and mice, several proteases such as caspase 1, elastase and cathepsin G are known to recognise specific sequence motifs in the central region of the proIL-1β protein, separating the mature peptide from the precursor. Caspase 1, also termed IL-1β converting enzyme (ICE), is the primary protease for processing IL-1β. However, fish IL-1β orthologues lack an identifiable ICE cut site [13,14,20,22,25,32,33,34,35]. Nevertheless, emerging evidence indicates that teleost IL-1β can be cleaved by caspases and processing of proIL-1β into the active form could involve canonical and non-canonical activation of effector caspases, notably caspase 1 and 8 within the inflammasome [22,29,36,37]. Various cut sites within a short region in the middle of the proIL-1β have been reported in different species [29,37,38]. Using a monoclonal antibody against IL-1β, a protein of 15 kDa, smaller than the theoretical size of the predicted mature protein, was detected in carp leucocyte cultures activated by PHA [39]. In the rainbow trout monocyte/macrophage cell line RTS-11 transfected with an IL-1β expression plasmid, an IL-1β protein of approx. 24 kDa is secreted into the culture medium [40]. Interestingly, zebrafish IL-1β can be cleaved into two peptides of 22- and 18-kDa by two distinct caspase 1 homologues after infection with a fish-specific bacterial pathogen *Francisella noatunensis*, indicating cleavage at multiple sites is possible and may depend on the immunological environment [29]. Direct evidence to support the involvement of caspase 1 came from a recent study showing that the seabass IL-1β can indeed be cut by recombinant caspase 1, resulting in a mature peptide of 18.5 kDa. Curiously, the identified cut site for the caspase 1 is located within the predicted first beta sheet of the mature peptide [37], and so it is not certain whether the processed mature IL-1β is biologically active. In contrast to these reports, cleavage of IL-1β in seabream macrophages is not affected by caspase-1 or pan-caspase inhibitors and is independent of caspase-1 [26]. In mammals, caspase 8 has been shown to directly cleave proIL-1β whilst caspase 11 is required for IL-1β proteolysis via activation of caspase 1 [41,42]. Some of these caspases have been characterised in fish and are shown to be important for regulation of the inflammatory response [38,43,44,45]. In addition, neutrophil elastase and some cathepsins may play a role in regulating maturation and release of active IL-1β, as their human counterparts are shown to be capable of cleaving proIL-1β [46,47]. Taken together, these observations suggest that the mechanisms for processing of fish IL-1β are complex and are tightly regulated during inflammation.

IL-1β has diverse physiological functions and its roles in regulating the inflammatory process are conserved in fish [1]. Although the native fish IL-1β proteins have not been purified, recombinant proteins have been produced in bacteria and in general are biologically active [13,32,34,48,49,50,51,52]. To date, most *in vivo* studies have been focused on the transient and local effects of IL-1β on the immune system. Intraperitoneal administration of IL-1β into trout increases the number of phagocytes that migrate into the peritoneal cavity and the phagocytic and lysozyme activity of macrophages [48]. The local effect of IL-1β is also apparent in the elevation of TNF-α and IL-1β expression in muscle tissue injected with an IL-1β encoding expression plasmid [52]. When administered directly into the intestine of grass carp, IL-1β induces severe gut inflammation and expression of TNF-α [32]. Fish IL-1βs also modulate expression of IL-17 family members, important for antibacterial defence [1,53,54]. In trout head kidney (HK) leucocytes, IL-1β up-regulates IL-17C2 but not IL-17A/F [55,56]. IL-1β has also been shown to enhance antibody production when administered with bacterial vaccines, suggesting it may be exploited as an immune-adjuvant for improving vaccine efficacy [13,52].

In a microarray analysis, Martin *et al.* [57] identified a panel of genes that respond to IL-1β in RTS-11 cells. In other studies, IL-1β action has been extensively analysed in primary leucocytes and macrophages where induced expression of pro-inflammatory genes is evident. Such genes include TNF-α [58], IL-1β [20,34], IL-6 [59], IL-8 [59], IL-34 [60], and cyclooxygenase -2 (COX-2) [34,51]. The induced expression of IL-6 and COX-2 by IL-1β can be inhibited by the stress hormone cortisol [59]. IL-1β has also been shown to activate expression of genes which are suppressors of the immune response. For example, trout IL-1β differentially modulates cytokine inducible Src homology 2 (SH2)-containing proteins (CISHs) and strongly enhances CISHa2 expression but interestingly not CISHa1 [61]. In addition, immune suppressive cytokines such as IL-10 and TGF-β1b can be induced by IL-1β in primary HK derived macrophages and RTS-11 cells [59,62]. The inducible effect of IL-1β on TGF-β1 appears to be mediated via the NF-κB and MAPK signalling pathways [63].

IL-1β is a chemoattractant for leucocytes in fish. Chemotaxis of leucocytes is coordinated by a sequential gradient of chemokines and the activation of G protein-coupled receptors. *In vitro* stimulation of freshly isolated trout leucocytes with IL-1β protein or peptides leads to enhanced cell migration [64]. This may be associated with the rapid release of intracellular Ca+ ions [65], as well as the up-regulation of the transcript levels of chemokine receptors on target cells [66]. In addition to its direct impact on target cells, IL-1β stimulation augments chemokine production in cells at infection sites. Several CXC chemokines, including CXCL8_L1 (IL-8), CXCL11_L1 (gamma IFN inducible protein, γIP), and CXCL_F4 and _F5 are increased in RTG-2 and RTS-11 cells after IL-1β stimulation [67].

In addition to its roles in immune regulation, IL-1β is involved in regulating other physiological processes. Recent studies demonstrate that fish muscle metabolism is affected by IL-1β. Trout primary muscle cells incubated with IL-1β exhibit higher expression of both inflammatory genes and genes related to muscle growth and metabolism [68]. In addition, IL-1β increases expression of atrogin-1, a key ubiquitin E3 ligase controlling muscle mass, and insulin growth factor binding protein (IGFBP) -6 in salmon muscle cells [69], and induces dilation of isolated steelhead trout coronary microvessels [70].

**Figure 1:**
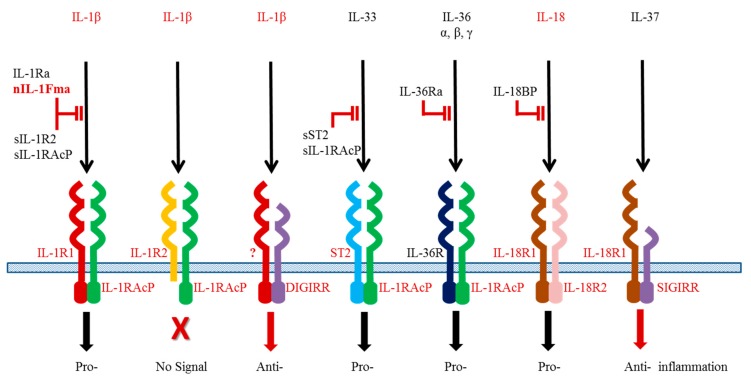
Interaction of the IL-1 family cytokines and receptors. The homologues identified in fish are indicated in red.

The IL-1β functions are controlled at different levels. Interaction of IL-1β with its heterodimeric receptor is pivotal to downstream signalling and dictates the outcome of the cellular responses. The IL-1β receptor complex comprises a ligand specific chain (IL-1R1 or IL-1R2) and a common chain (IL-1R accessory protein, IL-1RAcP) shared by different members of the IL-1 family [71]. All the receptors have a similar secondary structure containing 1–3 immunoglobulin (Ig) like domains in the extracellular region, which engage with the ligand (Figure 1). The IL-1R1 serves as an agonistic receptor upon activation by the ligand whilst the IL-1R2 acts as a decoy receptor to block ligand actions. The IL-1R2 has a short intracellular cytoplasmic region lacking the Toll/IL-1 receptor (TIR) homology domain and hence is unable to interact with adaptor proteins for signal transduction. Soluble receptors exist as negative regulators for blocking IL-1β signalling.

The first fish IL-1R homologue was identified as an IL-1R related protein in Atlantic salmon [72], and later confirmed as the orthologue of IL-1R1 [73]. It has a typical arrangement of three conserved Ig domains in the extracellular region. Like its mammalian orthologues, salmon IL-1R1 forms a heterodimer with its co-receptor IL-1RAcP. A soluble form of IL-1R1 has recently been identified in orange-spotted grouper [74]. It can be induced by high concentrations of IL-1β and is suggested to be a potential negative regulator to dampen excessive IL-1β effects.

Teleost IL-1R2 has a well-conserved protein structure as a decoy receptor for antagonising IL-1β functions, and contains three Ig domains in the extracellular region and a short cytoplasmic region lacking a TIR domain [75,76,77]. One exception is the report of a two Ig domain-containing IL-1R2 in seabream, which is probably due to a prediction error of the software used [75]. The seabream IL-1R2 is expressed at the cell surface of transfected human HEK293 cells, and has been shown to bind to IL-1β [75]. Direct interaction between IL-1β and soluble IL-1R2 has also been confirmed using recombinant proteins produced in bacteria [78]. As expected, the soluble IL-1R2 protein inhibits the effect of IL-1β to induce expression of inflammatory genes in grass carp primary HK cells [78]. At the transcript level, the IL-1R2 is expressed in all tissues of healthy fish and LPS stimulation or bacterial infection (*Vibrio anguillarum* and *Aeromonas hydrophila*) results in a significant increase of gene expression [75,78,79]. Interestingly, IL-1R2 expression is not altered in amoebic gill disease affected fish or down-regulated in fish with parasitic infections (*Ichthyophthirius multifiliis*) [75,76,80].

A novel double Ig IL-1R related molecule (DIGIRR) (Figure 1) has been added to the teleost IL-1R family [81]. It has a close phylogenetic relationship with the mammalian Single Domain IL-1R related (SIGIRR) molecule and may act as a negative regulator for inflammatory responses in fish. The anti-inflammatory activities of DIGIRR have been investigated in zebrafish where DIGIRR is able to abrogate LPS-induced and IL-1β-induced NF-κB activation when injected into embryos. When the DIGIRR expression is suppressed in fish by small interfering RNA molecules, expression of pro-inflammatory cytokines is elevated.

In addition to the receptors, the functions of IL-1 family cytokines are known to be controlled by multiple intracellular proteins. Upon activation of the receptors by the ligands, Myeloid differentiation factor 88 (MyD88) and Toll interacting protein (Tollip) are recruited to the IL-1R1/IL-1RAcP complex, resulting in phosphorylation of the IL-1R-associated kinases (IRAKs). The phosphorylated IRAKs interact with the TNF receptor associated factor (TRAF) 6 and NF-κB is then activated, leading to expression of pro-inflammatory genes in the target cells. To date, all these signalling molecules have been found in fish, suggesting that the IL-1 signalling pathway is well conserved among vertebrates.

### 2.2. IL-18

IL-18 is an important cytokine found initially to induce IFN-γ production and promote Th1 immunity in vertebrates [82,83]. As a member of the IL-1 family, it also plays an important role in regulating inflammation in mucosal tissues such as skin and gut. Akin to IL-1β, IL-18 is constitutively synthesised as an inactive precursor in healthy animals and is released after cleavage by caspase 1 following stimulation with PAMPs or DAMPs. Mature IL-18 binds to a heterodimeric receptor consisting of IL-18R1 and IL-18R2 (Figure 1). A soluble protein, termed IL-18 binding protein, is known to block binding of IL-18 to the receptors.

Compared with IL-1β, fish IL-18 has been under-investigated. To date, IL-18 has been reported only in a few fish species including rainbow trout [18], pufferfish [16], seabream [84], turbot [85] and elephant shark [86]. Fish IL-18 is encoded by a single gene comprising six exons and five introns, a conserved organisation seen for all known IL-18 genes [16,18]. Unlike IL-1β, which is temporally induced by inflammatory stimuli, IL-18 is constitutively expressed in fish immune or non-immune tissues [18]. Interestingly, IL-18 expression is not modulated by LPS, poly(I:C) or IL-1β in freshly isolated trout leucocytes [18]. However, a shorter variant of the alternatively spliced transcript (trout IL-18B) is upregulated in RTG-2 cells (a trout fibroblast cell line) after stimulation with LPS or poly(I:C) but not IL-1β. This work suggests that proteolytic processing of the inactive precursor may be crucial in mediating fish IL-18 functions which have yet to be characterised.

A partial IL-18 receptor (IL-18R1) has been sequenced in zebrafish [87]. A conserved TIR domain is predicted in the sequence. Constitutive expression is detected in healthy fish and is not affected by intraperitoneal injection with *Mycobacterium marinum* (*M. marinum*). However, an increase of IL-18R1 transcripts is observed in the intestine of seabream infected with *Enteromyxum leei*, a myxozoan parasite, suggesting that IL-18 may play a role in mucosal immunity [84]. The IL-18R2 has not been identified in fish.

### 2.3. Fish Specific Novel IL-1 Family Member

A novel IL-1 family member (nIL-1Fm) has recently been discovered and studied in a few fish species [20,88]. This gene(s) appears to be teleost specific and has ambiguous phylogenetic relationships with known IL-1 family members. Like most of the IL-1 family members, it lacks a signal peptide and has a pre-domain [17,20,22]. Sequence analysis indicates that conserved cut sites for caspase 1 and thrombin are present in the trout nIL-1Fm, suggesting proteolytic processing is required to generate the active protein. A recombinant trout nIL-1F protein comprising the last 169 aa of the C-terminal region has been produced in bacterial cells and is unable to trigger expression of inflammatory genes such as IL-1β, TNF-α, COX-2 and CXCL8_L1/IL-8 but inhibits the IL-1β-induced gene expression in RTS-11 cells, suggesting it acts as an antagonist of IL-1β [17]. The antagonistic effect of nIL-1Fm is likely mediated by competition with IL-1β for receptor binding, as shown in grass carp [88].

## 3. B-Jellyroll Cytokines

The jellyroll cytokine family, also referred to as the tumour necrosis factor superfamily, comprises 19 ligands (TNFSF) and 29 receptors (TNFSFR) in humans [89]. The three major members of this cytokine family are TNF-α, lymphotoxin (LT) -α (also called TNF-β) and LT-β. The TNFSF members are usually type II membrane proteins that contain a short N-terminal intracellular domain, a transmembrane domain and a C-terminal extracellular TNF homology domain with a conserved TNF family signature motif. An exception is LT-α that has a signal peptide and can be secreted conventionally. However, following enzymatic cleavage many of the mature peptides of the TNFSF can be released from the cell surface as soluble forms. Both TNF-α and LT-α are released as homotrimers that interact with the receptors on target cells. In addition, LT-α is required for the transport of LT-β to the cell surface to form a membrane bound LT-α/LT-β complex [90], and increasing evidence suggests that membrane bound ligands play a critical role in regulating the inflammatory response [91]. A secreted LT-α/LT-β heterodimer has also been described [92].

TNF is an ancient cytokine and functional homologues can be traced back to invertebrates [4]. In bony fish, TNF genes have been reported in a number of species [93,94,95,96,97,98,99,100,101,102,103,104,105], and their phylogeny has been studied extensively [106,107]. Evidence gathered to date demonstrates that multiple TNF homologues/paralogues exist, which can be categorised into three phylogenetic groups, the type I and II TNF-α group and the TNF-N group [58,108,109]. The genes encoding type I TNF-α and TNF-N are tandemly linked in a single chromosomal locus across the teleosts, exhibiting a conserved synteny with that in humans. In contrast, the type II TNF-α genes appear to be teleost specific [58,108]. Within the subfamily, multiple copies of type I or II TNF-α genes have been found in some species such as Atlantic salmon, rainbow trout, goldfish and common carp [58,97,104,110,111,112,113]. The recombinant proteins of fish TNF-α paralogues display similar bioactivities, indicating redundant functions in immune regulation. However, they are differentially modulated in different cell types and the kinetics vary, suggesting they do play different roles in the immune response [58,104,111,114]. For example, trout TNF-α3, a fish type II TNF-α, is implicated to be the major TNF-α isoform involved in T cell mediated immunity in addition to its roles in innate immunity [58,114]. In contrast to TNF-α, TNF-N is encoded by a single copy gene [58,100] and its functions have yet to be characterised.

Release of soluble TNF-α requires the removal of the precursor region, including the N-terminal intracellular domain and transmembrane region [91]. This process is facilitated by the TNF-α-converting enzyme (TACE, also referred to as ADAM17) which cleaves the proTNF-α at a specific site. Unlike fish IL-1βs where the ICE cut site region differs significantly, a predicted cut site for TACE is present in all fish TNF-α sequences, suggesting a conserved mechanism for TNF-α processing and release in all vertebrates [58,96,97,105]. Recently, TACE activity has been described in rainbow trout macrophages after stimulation with LPS, where it promotes the processing of proTNF-α [115]. The TACE/ADAM17 gene has also been confirmed in ayu, supporting the involvement of TACE/ADAM17 in release of TNF-α in fish [116].

As a pro-inflammatory cytokine, TNF-α is one of the early immune genes expressed at an early stage of infection in fish and has a key role in regulating inflammation. Like its mammalian counterparts, fish TNF-α displays overlapping functions with IL-1β. Many fish TNF-αs have been produced in bacteria as monomers, dimers and trimers and are able to activate macrophages/phagocytes and enhance their microbial killing activity [103,117,118]. *In vitro* treatment of primary trout HK leucocytes and monocytes/macrophages with TNF-α triggers expression of a number of immune genes associated with inflammation, including IL-1β, IL-8, IL-17C, TNF-α and COX-2, and genes involved in antimicrobial responses [58,103,105,119]. The stimulatory effect of TNF-α involves the NF-κB signalling pathway [58,103,119,120]. For example, incubation of grass carp leucocytes and EPC cells with TNF-α results in a rapid increase of NF-kB activity in the treated cells [103,120].

The TNF-α protein enhances the phagocytic activity of fish leucocytes [105,110,117]. In *M. marinum*-infected zebrafish, TNF-α has been shown to promote macrophage survival and also restrict bacterial growth in infected macrophages [121]. The bacterial-killing activity appears to be accompanied by increased generation of reactive oxidative species (ROS) [116,122]. However, in the case of zebrafish infected with *M. marinum*, excessive production of TNF-α-activated ROS is detrimental to the host cells, leading to programmed necrosis and increased release of mycobacteria from the infected macrophages [122].

Fish TNF-αs are suggested to be involved in the regulation of leucocyte homing, proliferation and migration. In trout thymus, constitutive expression of TNF-α is detected, suggesting it could play a role in promoting thymocyte growth [123]. Trout TNF-αs also increase migration of enriched primary HK macrophages in a dose-dependent manner [105,117], and an *in vivo* experiment shows that intraperitoneal injection of seabass with TNF-α results in rapid recruitment of phagocytic granulocytes to the peritoneal cavity [117]. In addition, TNF-α activates chemokine expression in local tissue cells such as endothelial cells in zebrafish and gilthead seabream [124]. Another study shows that the supernatants from TNF-α-treated endothelial cells can promote leucocyte migration and respiratory burst activity in carp [125]. In the zebrafish model, a cellular adaptor protein termed factor associated with neutral sphingomyelinase activity (FAN) has been shown to co-ordinate TNF-α activated chemotaxis in response to infection and wounds [126].

Fish TNF-αs exert pro-apoptotic activity, as seen with their mammalian homologues. High doses (400–4000 ng/mL) of Chinese perch TNF-α induce apoptosis of human Hela cells [102]. In tilapia TNF-α has been shown to upregulate granzyme expression in non-specific cytotoxic cells and provides protection of these cells from activation-induced cell death [127]. These results suggest that TNF-α triggered apoptosis is likely to be dose and target cell dependent.

In addition to its roles in acute infection, accumulating evidence suggests that TNF-α is associated with pathogenesis of several chronic diseases in fish. TNF-α, together with other pro-inflammatory genes, is induced in the heart during pathology of salmonid pancreas disease [128]. In turbot, during infection with *Enteromyxum scophthalmi*, the increased number of the TNF-α+ cells leads to the infiltration of inflammatory cells in the intestine and is associated with the development of the lesions, epithelial shedding and intestinal barrier dysfunction [129]. Recently, Marjoram *et al.* [130] studied TNF-α production in the gut during infection with *M. marinum* using transgenic TNF/GFP reporter fish, and found strong up-regulation of TNF-α in granulomas surrounding the bacteria. The unregulated induction of TNF-α expression in intestinal epithelial cells (IECs) exacerbates gut inflammation, resulting in IEC shedding and apoptosis, immune cell recruitment, and barrier dysfunction of the gut in zebrafish [130]. Lastly, TNF-α is required for oocyte maturation in fish [131,132], and is essential for liver development in zebrafish [133], where knockdown leads to a reduction of liver size [133].

## 4. Cysteine Knot Cytokines

The cysteine knot cytokines get their name from a disulphide-rich, all-beta core domain protein secondary structure. They consist of two families of particular importance for immunity, namely the IL-17 family cytokines and the transforming growth factor-β (TGF-β) family cytokines. However, IL-17 family members contain only two of the three characteristic disulphide linkages that give the family its name [134]. They are ancient cytokines with TGF-β superfamily members and IL-17 homologues present in invertebrates. In addition, in invertebrates Spätzle, an endogenous Toll receptor ligand [135], has a cysteine knot structure and is critical for the initiation of innate immune responses in molluscs and arthropods.

### 4.1. Interleukin-17

Six IL-17 family members exist in mammals, IL-17A to IL-17F. However, in fish, only two clear homologues are known, IL-17B and IL-17D, with multiple isoforms of molecules termed IL-17A/F (IL-17A/F1-3, and IL-17N) with relatedness to both IL-17A and IL-17F, and IL-17C with relatedness to IL-17C and IL-17E (IL-25) being present [4,56,86,136]. Relatively few studies have reported the bioactivity of any of these IL-17 molecules in fish. In grass carp rIL-17A/F1 can increase IL-1β, IL-6, IL-8 and TNF-α expression in HK leucocytes [137], and in trout rIL-17A/F2a increases the expression of IL-6, IL-8 and an antimicrobial peptide (β-defensin 3) in splenocytes [138]. In the case of grass carp rIL-17D, it can also increase IL-1β, IL-8 and TNF-α expression in HK cells but does not increase IL-6 expression [139], hinting at subtle differences in the ability of different isoforms to induce pro-inflammatory genes.

In relation to the possible sources of the IL-17 isoforms in fish, such as potential Th17 cells or group 3 innate lymphoid cells (ILC), it has been shown that enhanced IL-17A/F2 expression is seen in CD4-1+ lymphocytes isolated following specific antigen-restimulation of zebrafish three days earlier [140]. Th17 cell development is inhibited by Treg cells that are driven to differentiate by the transcription factor FoxP3, and also in zebrafish it has been shown that injection of zebrafish eggs with a plasmid encoding FoxP3 results in inhibition of IL-17 (isoform not given) expression in six-day-old embryos [141]. In contrast injection of eggs with morpholinos to inhibit FoxP3 translation results in up-regulation of IL-17 in five- day-old embryos.

### 4.2. Transforming Growth Factor-β

TGF-β also has multiple isoforms, TGF-β1 to TGF-β3, and there appear to be true homologues of these genes throughout vertebrates (note: Chicken TGF-β4 = TGF-β1; Xenopus TGF-β5 = TGF-β1). However, a fish specific TGF-β isoform (TGF-β6) has been reported [142] although the role of this molecule remains to be determined. In the context of immune responses, most studies have focused on TGF-β1 function as a molecule known to drive Th17 differentiation in combination with other cytokines (eg IL-6) but which is also a key immunosuppressive cytokine secreted by Treg cells. TGF-β1 has been produced as a recombinant protein in several fish species, and shown to have an immunosuppressive function. For example, in goldfish rTGF-β1 can inhibit the nitric oxide response of TNF-α activated macrophages [143], and in grass carp rTGF-β1 inhibits the LPS induced up-regulation of TNF-α, IL-1β, IL-8 and inducible nitric oxide synthase (iNOS) in monocytes/macrophages [144]. In the latter case incubation in the presence of an antibody to TGF-β1 increases these transcript levels. Further studies have shown that grass carp rTGF-β1 is able to inhibit the up-regulation of IL-1β (transcript and protein) by rIL-1β in HK leucocytes but has no effect on constitutive IL-1β levels [63]. In addition, grass carp rTGF-β1 decreases the basal level and IL-1β induced up-regulation of the IL-1 agonistic receptor chains (IL-1R1 and IL-1RAcP) but enhances the expression of IL-1R2, the decoy receptor (Figure 1). These effects appear to be mediated by impaired IκB phosphorylation but increased phosphorylation of Smad2. Co-incubation with the TGF-βRI kinase inhibitor VIII (ALK5 inhibitor) has also been shown to ablate rTGF-β1 action in grass carp, indicating that TGF-β1 has a self-regulatory function in fish [145].

rTGF-β1 can also enhance the proliferation and viability of peripheral blood leucocytes (PBL) [145,146] and the proliferation of fibroblasts [143] but reduces HK cell viability and blocks the proliferation of PBL induced by PHA or LPS [145,146]. It also suppresses erythropoiesis [147]. Indeed, there is an increased level of (active) TGF-β1 protein and up-regulation of TGF-β signalling in the Antarctic notothenioid fish that lack hemoglobin/erythrocytes, compared to red-blooded notothenioids. TGF-β1 protein has also been detected in tilapia, where it was reported to increase from 12 h to three days in the HK, and from one to three days in spleen post-injection with *Streptococcus agalactiae* [148].

## 5. Type I α Helical Cytokines

The type I helical cytokines include the bulk of the interleukins, and the colony stimulating factors. The former can be subdivided into the IL-2 subfamily, the IL-6 subfamily and the IL-12 subfamily, with the IL-2 subfamily considered short type 1 cytokines and the IL-6/12 subfamilies long chain type I cytokines. They signal via receptors of the type I cytokine receptor family [149]. Representatives of all these cytokine families are present in fish, although rather few of the proteins have had their bioactivity analysed.

### 5.1. IL-2 Subfamily

The IL-2 subfamily includes IL-2, IL-4/13, IL-7, IL-15 and IL-21 in fish. The IL-4/13 molecules have relatedness to both IL-4 and IL-13, and in teleost fish have diverged into IL-4/13A and IL-4/13B due to the duplication of the locus from the whole genome duplication (WGD) event at the base of this fish lineage [150,151]. IL-2 has been made as a recombinant protein in rainbow trout, where it has been shown to increase the expression of STAT5 and Blimp-1, transcription factors involved in IL-2 signalling, IFN-γ, the chemokine γIP, and itself in HK leucocytes [152]. Later, it was shown that rIL-2 can also up-regulate the expression of the fish type-2 cytokines (see below) IL-4/13B1 and IL-4/13B2 (in HK cells) but had no effect on IL-4/13A [153]. In addition, a small increase of IL-17A/F1b and IL-17A/F2a expression was seen after stimulation of HK cells [56]. A stable trout cell line has been made that over-expresses IL-2, and the conditioned media (CM) harvested for establishing IL-2 dependent cell lines [154]. When HK cells were incubated with these CM for 3–5 weeks a major shift to a dominance of CD4-1 transcripts (*vs.* CD8, IgMH and MCSFR) was seen in the cultures. However, if the cells were first treated with the T cell mitogen PHA, the CD8 transcripts were more highly represented (26%–51%) in addition to CD4-1 and MSCFR at these times.

In the case of IL-4/13 bioactivity, initial studies looked at the *in vivo* effects of injecting the protein into fish, and found that in zebrafish IL-4/13A could increase the number of CD209+ (DC-SIGN) cells in PBL five days post-injection [155], the number of IgZ-2+ B cells in PBL at two days post-injection [156], and the number of IgM+ B cells in PBL at three days post-injection [157]. rIL-4 administration also increased the transcript expression of mIgM, MHC II and CD80 in these cells at the latter timing. In addition, injection of rIL-4 (×3 at 12 h intervals) prior to immunisation gave higher specific serum antibody titres compared to fish given antigen (KLH) alone. The zebrafish recombinant IL-4R has also been shown to bind IL-4/13A in a pull-down assay, and *in vivo* administration of the rIL-4R soluble form or an anti-IL-4Rα Ab (for receptor blockade) ablates the *in vivo* effects of rIL-4 described above [157]. Most recently the activity of rIL-4/13B has been studied. In carp, rIL-4/13B stimulates the *in vitro* proliferation of IgM+ B cells, in terms of colony formation and increased cell number [158]. In rainbow trout rIL-4/13B (IL-4/13B2, one of the two paralogues of this molecule present in trout) was studied alongside rIL-4/13A, and these cytokines were found to have shared but also distinct activities in terms of transcriptional modulation of other immune genes [153]. In addition, both cytokines increased the number of IgM secreting cells (likely by inducing the transcript for the secretory form of IgM rather than the membrane form) but had no effect on their proliferation, in contrast to the situation in carp. Taking into account the different expression patterns of these two genes in trout, it was proposed that IL-4/13A may be important for basal type-2 immunity, whilst IL-4/13B expression may aid adaptive responses post-infection/immunisation in this species.

Whilst IL-7 is known in fish [159] there have been no functional studies using the recombinant protein. However, in zebrafish it has been shown that fish with a mutation in the IL-7R, have impaired thymopoiesis [160]. Both ikaros-expressing and rag1-expressing cells are reduced in the thymus of such fish. The fish tend to die early but those that survive have a smaller and hypocellular thymus relative to that of wild type fish. However, the HK is unaffected, with numbers of IgMH chain expressing (B) cells being normal. The mutation arises from an insertion of an extra thymidine residue in exon 4 that induces a frameshift that impacts on the extracellular domain and the truncated protein, if produced, is predicted to be unable to interact with IL-7. These results support the paradigm that IL-7 signalling is required for T cell development throughout the vertebrates.

IL-15 has been made as a recombinant protein in fish, in rainbow trout, and its bioactivity studied [161]. Initially it was shown to increase IFN-γ expression in primary cultures of splenocytes but had no effect on HK cells or the RTS-11 (macrophage) cell line. Later it was also shown to increase expression of two of the IL-12 family peptides (see below), p35 (two fold) and p40c (six fold), in HK cells but not p40b expression, the second p40 paralogue present in trout [162]. Studies in mammals suggest that IL-15 is pleiotropic with effects on many cell types. Thus, it remains to be determined if these relatively restricted responses are found in other fish species. A second IL-15 related gene that has also been identified in fish is located at a different locus to the IL-15 gene [163,164], and has been termed IL-15-like (IL-15L). It was subsequently found to be present in other vertebrate groups but is a pseudogene in man and mouse [165]. Functional studies have still to be performed with this molecule, which in mammals binds to the IL-15Rα but not the IL-2Rα, as seen with IL-15 itself [165].

In contrast to IL-15, rainbow trout rIL-21 has been made and shown to have a wide range of effects [166]. When added to HK cell cultures it was able to maintain the expression level of several T and B cell markers, suggesting it is likely an important lymphocyte survival factor. It also up-regulated the expression of several key cytokines and immune regulators, including IL-6, IL-10, IL-22, IFN-γ, γIP, STAT3, STAT5, Suppressors of Cytokine Signalling (SOCS) 1, SOCS3 and itself. In addition, *in vivo* administration (intraperitoneal injection) of rIL-21 induced the expression of IL-10, IL-21 and IFN-γ in peritoneal adipose tissue, and IL-10 and IL-22 in the HK at Day 1 post-injection, and IgMH and CD8 in the HK at Day 3. Subsequent studies have shown that rIL-21 can also increase p40c expression (but not p35 or p40b) [162], IL-17A/F1a/b and IL-17A/F3 expression [56], and IL-4/13A (in contrast to IL-2)/IL-4/13B (albeit relatively late compared to IL-2) expression in HK cells [153]. The rIL-21 induced up-regulation of IFN-γ in HK cells was ablated by pre-treatment with Stattic, a STAT3-specific inhibitor, and was partially reduced by pre-treatment with Jak inhibitors [166]. Such findings suggest that these IL-21 induced effects are mediated via a Jak/STAT3 pathway.

These cytokines signal via receptors that contain the common gamma chain (γC) CD132 as a shared subunit with at least one private chain. In some fish species two γC genes are known, as in zebrafish where they are termed γC.a and γC.b. Gene knock-down of γC.a with anti-sense morpholinos results in significantly lower embryonic lymphopoiesis and loss of mature T cells in the larvae, whilst mature B cells could still be detected similar to the situation with SCID mice [167]. This was not seen with knock-down of γC.b, and its function remains to be determined. Receptor chains similar to the known private chains are present in fish [168] with the apparent exception of the IL-2Rα. It has been suggested that in fish IL-2 and IL-15 may both bind to the IL-15Rα (also termed CD25-like in some papers), although a 5-fold preference for IL-15 was seen in binding assays with the Tetraodon molecules [169]. Currently it is unknown whether IL-2Rα evolved after the divergence of bony fish from the tetrapod lineage or whether it has been secondarily lost in these fish [165].

### 5.2. Beta Chain Cytokines

The beta chain (βc) cytokines include IL-3, IL-5 and GM-CSF, and signal via a receptor composed of the beta-chain (or CD131) and a cytokine specific private chain. The beta chain has been known in fish for some time [170] but until recently no obvious ligands had been detected. However, with the release of the elephant shark genome Dijkstra [171] reported the presence of two possible “IL-5 family” genes, and has subsequently found further possible IL-3/IL-5/GM-CSF related genes in spotted gar and in several cyprinid fish species [172]. The true nature of these genes remains to be determined and to date there are no functional studies.

### 5.3. IL-6 Subfamily

The IL-6 subfamily of cytokines are known to be major players in haematopoiesis, and have pro- and anti-inflammatory properties In fish four members of the IL-6 family exist; IL-6 itself, IL-11, a molecule termed CNTF-like and M17 [173,174]. Phylogenetic analysis suggests that the fish CNTF-like and M17 genes may have arisen from ancestral genes that gave rise to mammalian CNTF/CLC/CT-1/CT-2 and LIF/OSM, respectively. IL-31, a further member of the IL-6 cytokine subfamily in mammals, is unknown in fish to date. As with other fish cytokines, the fish IL-6 subfamily genes can have multiple paralogues, as seen with IL-11 [175,176].

No bioactivity studies have been reported for fish IL-11 or CNTF-like to date. However, IL-6 and M17 have been made as recombinant proteins. The rIL-6 has been shown to have effects on macrophages and B cells. In rainbow trout rIL-6 promoted macrophage growth in culture, with numbers of (RTS-11) cells being significantly increased after 4–7 days of treatment [177]. In addition the trout rIL-6 induced transient expression (up to 4 h post-stimulation) of SOCS1-3 and IFN regulatory factor (IRF)-1 but more sustained up-regulation of antimicrobial peptide (AMP) gene expression in these cells, with similar results seen with primary (HK derived) macrophage cultures. Interestingly, whilst the AMP cathelicidin-2 was sensitive to rIL-6 stimulation, cathelicidin-1 was unresponsive. STAT3 was shown to be phosphorylated following rIL-6 stimulation of RTS-11 cells, and an inhibitor of Jak2 (WP1066) could abolish the phosphorylation and inhibit cathelicidin-2 up-regulation, suggesting the JAK2/STAT3 pathway plays an important role in IL-6 signalling. rIL-6 was subsequently shown to increase IL-17A/F3 in HK cell cultures, with no effects apparent on the other IL-17A/F isoforms including IL-17N [56].

In terms of effects on B cells, CM from a stable cell line (RTG-2 cells) over-expressing trout IL-6 were found to maintain cells expressing the IgMH transcript in HK primary cultures for up to five weeks [154]. This was verified by FACS analysis of the numbers of IgM+ (B) cells present one week after cell isolation, in comparison to cells not receiving the IL-6 CM. In orange-spotted grouper rIL-6 was injected into fish, at 50 ng/g fish, and shown to increase the expression of IL-6, a number of transcription factors involved in T cell differentiation (Tbet, GATA3, c-maf) as well as IgMH, with effects most pronounced in the HK but also apparent in the trunk kidney and spleen [178]. Interestingly, whilst Tbet was initially increased, unlike GATA3 and c-maf it was subsequently suppressed (until 360 h post-injection) relative to PBS injected fish, hinting at a preferential effect of IL-6 on Th2 pathways. In addition, serum total and specific IgM was found increased post-injection, with total IgM levels elevated from Weeks 1 to 6 (the end of the study) and specific IgM against BSA increased from Weeks 2 to 6, in a dose dependent fashion (50 ng/g to 200 ng/g rIL-6 injected). Lastly, in fugu it has been found that IgM+ B cells (from PBL) express the two IL-6 receptor chains (IL-6R and gp130), and that stimulation of these cells with rIL-6 increased the expression of the secretory form of IgMH [179]. As with trout macrophages, rIL-6 stimulation resulted in STAT3 phosphorylation in these IgM+ cells and in Ba/F3 cells (a murine IL-3 dependent pro-B cell line) co-transfected with fugu IL-6R and gp130. The potential for IL-6 trans-signalling in fish, as occurs in mammals, was also verified with the discovery of a soluble form of the IL-6R in this species, that may permit signalling in cells expressing only gp130.

Goldfish M17 also affects macrophage function. Cells treated with M17 release nitric oxide and this effect can be increased in the presence of LPS [180]. Whilst M17 does not affect the proliferation of macrophages/monocytes/progenitor cells by itself, in the presence of CM from kidney macrophage primary cultures an enhanced proliferation was seen in the progenitor cell population. M17 also induces the differentiation of monocytes into macrophages but has no effect on progenitor cells. Such results confirm M17 as an important cytokine for macrophage proliferation, differentiation and activation in fish. In zebrafish knockdown of M17 (LIF) *in vivo* delays functional recovery after optic nerve injury, showing it also has a role in the nervous system [181].

### 5.4. IL-12 Subfamily

The IL-12 subfamily of cytokines consist of heterodimers of an alpha chain (p19, p35, p28) with structural similarity to the IL-6 family ligands, and a beta chain (p40, EBI3) with similarity to the extracellular domain of IL-6 family receptors. To date four members are known, namely IL-12 (p35/p40), IL-23 (p19/p40), IL-27 (p28/EBI3) and IL-35 (p35/EBI3). Whilst engagement with their receptors is unknown it can be modelled based on the IL-6-IL-6R structure [182]. In addition, p40 can form homodimers, that can act as an IL-12 antagonist [183]. All of these chains are known in fish and in many cases multiple paralogues are present, as seen in salmonids where there are ×2 p19, ×2 p28, ×3 p35, ×3 p40 genes and a single EBI3 gene [4]. 

To date only IL-12 has been studied functionally in fish, and in one study two different paralogues of the p40 chain were used to make two proteins with a common p35 chain [162,184,185]. In grouper rIL-12 was able to increase the Con A induced proliferation of PBL, PBL migration and TNF-α gene expression [185]. p40 alone was also able to induce chemotaxis and TNF-α expression but to a lesser extent compared to IL-12, and when added to the rIL-12 reduced its activity. When delivered orally as chitosan nanoparticle encapsulated IL-12 (at a dose of 20 μg/g fish) grouper survival was prolonged following challenge 20 days later with *Cryptocaryon irritans* theronts. In rock bream, rIL-12 pre-incubation (for 1 h) was shown to enhance the respiratory burst of PBL induced by *Vibrio alginolyticus* [184]. *In vivo* administration of rIL-12 (intraperitoneal injection, 20 μg/fish) for 4 h resulted in increased expression of several immune genes in kidney, including IL-1β, IFN, CC chemokine 1 (CCK1), IRF4, IFN stimulated gene (ISG) 15 and C7. Following injection with *V. alginolyticus* at this time lower CFUs were recovered from the kidney at Days 1 and 2 post-infection compared to fish given saline and protein controls. In rainbow trout two rIL-12 proteins have been made and shown to have overlapping but distinct bioactivity on HK cell cultures. The p35/p40b protein induced IFN-γ1 and IFN-γ2 expression, and p40b and p40c expression. However, a protein consisting of p35/p40c had a faster effect on IFN-γ1/2 (*i.e.*, induced significant effects at 6 h *vs.* 24 h for p35/p40b) and p40c expression (6 h *vs.* 48 h for p35/p40b) and in addition to modulating the above genes could induce IL-10 expression. These effects may reflect direct and indirect effects on gene expression mediated by p35/p40c and p35/p40b, respectively. Whether these proteins signal via different receptors remains to be determined but the p40 paralogues share only 24% aa identity and p40c appears to be preferentially induced by viral (viral haemorrhagic septicaemia virus, VHSV) and parasitic (proliferative kidney disease, PKD) infection in this species.

### 5.5. Colony Stimulating Factors

Colony stimulating factors (CSF) drive the proliferation and differentiation of blood cells from haematopoietic stem cells (HSC). Three CSFs are important for phagocyte differentiation from the common myeloid progenitor (CMP) in mammals, termed CSF-1 to CSF-3. CSF-1, also called macrophage colony stimulating factor (M-CSF), is a regulator of the mononuclear phagocyte lineage. It elicits its effects on the myeloid lineage by binding to the CSF-1R (CD115), as does the related molecule IL-34 which can additionally form a heterodimer with M-CSF for receptor docking [186]. Multiple splice variants of M-CSF exist in mammals that can have different effects on target cells. M-CSF was first described in fish from goldfish [187]. The recombinant protein was found to form a homodimer and to bind to the soluble goldfish CSF-1R (sCSF-1R), previously characterised in this species [188]. The goldfish rM-CSF was also shown to induce the differentiation of monocytes into macrophages and the proliferation of monocyte-like cells, in line with having an important role in mononuclear phagocyte function in fish, and this could be inhibited by addition of the sCSF-1R or an antibody to the CSF-1R [189]. Continuous addition of rM-CSF to goldfish primary macrophage cultures extended their longevity and resulted in the long-term cultures of functional macrophages [190]. In addition, stimulation of goldfish macrophages with rCSF-1 elevated their respiratory burst and nitric oxide responses, phagocytic activity and locomotion; all of which were inhibited by addition of sCSF-1R. The rM-CSF treated macrophages showed increased expression of immune genes such as CXCL8, CCL-1, TNF-α, IL-1β, IL-10, p35, p40, type I IFN, and iNOS [189], indicating activation of the cells. That the goldfish M-CSF acts through the CSF-1R was demonstrated by loss-of-function studies, where knockdown of CSF-1R in monocytes using RNAi abrogated monocyte proliferation and the differentiation of monocytes into macrophages [190]. *In vivo* administration (25 ng) of the rM-CSF two days earlier resulted in increased numbers of circulating monocytes (34% to 47%), and using BrdU to analyse cells undergoing DNA synthesis it was found that the labelled cells increased from 1% to 13% post-injection [190]. Lastly, supernatants from the goldfish fibroblast cell line CCL-71 can also induce the proliferation of primary macrophage cultures, and this effect is inhibited by addition of sCSF-1R, suggesting that fibroblasts in fish secrete M-CSF as seen in mammals [190].

Subsequent analysis of several teleost fish species revealed that in fact two major forms of M-CSF exist, that were termed M-CSF1 and M-CSF2, that likely arose from the teleost WGD event [191]. The two forms differ in size (M-CSF1 being larger and M-CSF2 shorter) but share a similar domain structure. The difference in protein size is due to variation in the region between the CSF-1 domain and transmembrane domain. Similar to the results with the goldfish molecule, rainbow trout rM-CSF1 was shown to be a growth factor for macrophages, and when added to macrophages or newly adherent HK cells could increase CXCR3 expression, as well as CSF-1R expression in the latter case. However, in contrast to the results with goldfish rM-CSF, trout rM-CSF1 had no effect on the expression of a variety of pro-inflammatory genes, including IL-1β, IL-11, IL-15, IFN-γ and TNF-α. As with IL-2/IL-6 above, a stable cell line (RTG-2 cells) over-expressing trout M-CSF1 has been developed and CM from these cells increases MCSFR2 relative expression in HK cells, such that by five weeks of treatment MCSFR2 transcripts represent 70% of those analysed (CD4-1, CD8a, IgMH, MCSFR2) [154]. This suggests that mononuclear phagocytes were the dominant cells being maintained by these M-CSF1 containing CM. No bioactivity studies with the teleost M-CSF2 have been performed to date. Similarly, whilst IL-34 has been discovered in several teleost species, to date no bioactivity studies have been performed, although differential expression of the three possible CSF-1R ligands suggest functional differences are likely present [192,193]. In mammals, IL-34 specifically directs the differentiation of myeloid cells in the skin epidermis [194].

CSF-2, also called granulocyte-macrophage CSF (GM-CSF), is discussed above under the beta-chain cytokines and it is possible an ancestral gene with relatedness the βc cytokines exists in fish although bioactivity studies are lacking. However, CSF-3 or granulocyte-CSF (G-CSF) has been discovered and studied functionally in fish. Most studies have been performed in zebrafish, where knock-down of the G-CSFR with morpholinos and overexpression of G-CSF by injection of eggs with *in vitro* transcribed G-CSF mRNA has been used to determine G-CSF function [195]. Such experiments show that there is an increase in G-CSFR+ cells and lyz+ cells (in lyz::EGFP fish) when G-CSF is used, but that these numbers fall following injection with the G-CSFR morpholinos. Importantly no increases were seen when both were co-injected, showing a likely ligand-receptor dependence. Whilst no effects on myeloperoxidase (mpo)+ cells (monocytes/macrophages) were apparent in the posterior regions of the embryo following injection with G-CSF mRNA, the anterior region population did increase and injection of G-CSFR morpholinos reduced the total mpo+ population. These findings suggest that G-CSF acts more generally on myelopoiesis rather than just affecting granulopoiesis. Further studies in zebrafish, on thrombopoiesis, showed that when thrombopoietin (Tpo) and erythropoietin (Epo) are added together to haematopoietic stem and progenitor cells (HSPCs), mixed colony forming unit thrombocytic erythroid cell (CFU-TE) colonies are found, whilst Tpo alone gave rise to CFU-T colonies, suggesting there is a common bipotent thrombocytic/erythroid progenitor (TEP) [196]. However, when G-CSF is added together with Tpo and Epo to the HSPCs, colonies derived from blood progenitors upstream of the TEPs are found, such as CMP cells, multipotent progenitor cells (MPPs) and haematopoietic stem cells (HSCs).

It is now known that two paralogues of the G-CSF gene are present in teleost fish, where in zebrafish one is located on chromosome 12 (G-CSFa or Gcsf-Chr12) and one on chromosome 19 (G-CSFb or Gcsf-Chr19). Both have now been shown to signal via the G-CSFR using loss- and gain-of-function experiments in zebrafish similar to the above [197]. Both paralogues have been made as recombinant proteins and shown to stimulate granulocyte and monocyte/macrophage differentiation in whole kidney marrow cells cultured *in vitro*, and generate myeloid colonies. However, G-CSFa was more potent at higher concentrations *in vitro* and participates in emergency myelopoiesis *in vivo*, whilst G-CSFb is active at lower concentrations *in vitro* and affects neutrophil migration to wounds, with knock-down of G-CSFb decreasing the percentage of neutrophils that arrive at the wound site via blood vessels [197,198]. Interestingly it was the numbers arriving after 1 h that were reduced, in agreement with studies showing other factors such as hydrogen peroxide are more important in the early stages of the inflammatory process [199].

## 6. Type II α Helical Cytokines

The type II α-helical cytokines include the IL-10 subfamily (IL-10, IL-19, IL-20, IL-22, IL-24, IL-26 and IFN-γ) and the type I and III IFNs, that are considered to be derived from a common ancestor [200]. They signal via receptors of the type II cytokine receptor family [149]. Various members of the IL-10 subfamily and type I IFNs exist in fish as outlined below, with the IFNs being particularly well studied (Table 1).

### 6.1. Type I IFN

Type I IFNs have diverse immune functions, including activation of antiviral defences and anti-proliferative effects. Type I IFNs have now been identified in all the jawed vertebrate lineages [86,201,202,203,204,205,206]. As in mammals, teleost fish possess two or four cysteine-containing subgroups, namely group I and II IFNs, which can be further classified into six groups phylogenetically (IFN-a-f) [207,208,209,210,211]. Group I IFNs (IFN-a, -d and -e) are ubiquitously present whilst group II IFNs (IFN-b, -c and -f) appear to be restricted to certain species [211]. Both groups have a ternary protein structure of four α-helices, which are conserved for the type II α-helical cytokines [212]. A notable CAWE motif is present at the C-terminus of group II IFNs but seems less apparent for group I IFNs [210].

Fish IFN genes are clustered in the genome, at loci with conserved gene synteny [207,209,211]. In zebrafish two loci are found, on chromosomes 3 and 12, which harbour three and one IFN genes, respectively. The open reading frame of all teleost fish type I IFN genes is separated by four phase 0 introns [202,207,209,213,214,215], in common with amphibian genes and unlike amniotes where the genes are intronless. The number of IFN genes in fish varies significantly among species, with salmonids having the highest gene copy number in all the jawed vertebrates [207,209,211,216].

### 6.2. Group I IFNs

IFN-as were the first group of type I IFNs sequenced in fish [201,217,218], and are now known to be present in siluriformes [216,217], cyprinids [201,207], and salmonids [209,210,211,218]. Most recently, the IFN-a genes have also been identified in medaka and turbot, suggesting they are likely more widely distributed in teleosts than previously thought [219,220]. The IFN-as are ubiquitously induced in fish cells after viral infection or activation by viral PAMPs, to some degree mimicking the expression pattern of IFN-β in mammals, and have been shown to up-regulate a large panel of ISGs which are essential for host antiviral defence [201,207,210,214,218,219,221,222]. The common ISGs responsive to IFN-as include Mx [201,210,218,221], viperin [207], ISG15 [223], IRFs [224,225], and grass carp reovirus induced genes (Gigs) [226]. Furthermore, trout IFN-a2/IFN2 increases the expression of MDA-5 and LGP-2, which are important intracellular PRRs for recognition of viral PAMPs, leading to the magnification of the IFN response [227]. The positive regulation of the IFN response by IFN-a is also observed in RTG-2 cells, where IFN-a stimulation significantly increases the expression of IFN isoforms including itself [208]. Several studies have examined the global impact of IFN-as on gene expression at the transcript level in cell lines [228,229], and in general, the genes affected by IFN-a are similar to what is observed in mammals. Interestingly, a recent study indicates that the black carp IFN-a is glycosylated at Asn (N)38 when produced in EPC cells but that glycosylation does not contribute directly to the antiviral property [230].

In salmon TO cells, the IFN responsive genes are significantly different from those modulated by Salmonid Alpha Virus (SAV), that has a positive single stranded genome. IFN-a1 enhances resistance of host cells against some but not all virus types. For example, IFN-a1 is effective in enhancing cell protection against SAV and Infectious Pancreatic Necrosis Virus (IPNV, a double-stranded RNA virus) but surprisingly is relatively ineffective against Infectious Salmon Anaemia Virus (ISAV, a single-stranded RNA segmented orthomyxovirus) in TO cells despite transient induction of antiviral genes [128,231,232,233]. The timing of IFN stimulation is critical to protection. When salmon TO cells are pre-incubated with IFN-a prior to infection (4–24 h) with SAV they are protected against virus-induced CPE but if administered at the time of infection and up to 24 h post-infection they are not [234]. In agreement with the *in vitro* studies, intramuscular injection of an expression plasmid encoding IFN-a1 into Atlantic salmon fails to increase survival against ISAV infection [223]. The mechanism(s) underlying the variable efficacies of IFN elicited protection against different viruses is currently not understood. However, it is known that viruses exploit diverse strategies to counteract host antiviral defences including the IFN-mediated pathway, and that strains of different virulence (as seen with virulent strain ISAV7 *vs.* avirulent strain ISAV4) vary in their activation of the IFN response [233].

Several studies suggest that IFN-a may play a role in the regulation of inflammation in fish. Zebrafish IFN-a induces expression of several pro-inflammatory genes *in vivo* when the recombinant protein (produced in HEK 293 cells) is injected into fish [235]. Turbot IFN2 (a member of the IFN-a group) has recently been characterised functionally. Surprisingly, when administered by intramuscular injection, it is unable to trigger an antiviral response and provide protection against VHSV [220]. However, unexpectedly, it has a stimulatory effect on the expression of pro-inflammatory genes such as IL-1β. Based on the fact that turbot IFN2/IFN-a is up-regulated during infection with VHSV and the bacterial pathogen *Aeromonas salmonicida*, it is suggested that turbot IFN2/IFN-a may also serve as a mediator in regulating inflammation.

In addition to the secreted IFN-as, transcript variants that translate into proteins that lack a signal peptide have been described in several teleost species [217,236,237]. Further analysis in trout reveals that such transcripts originate from mRNA alternative splicing of the intron(s) located at the 5’ end of the IFN-a gene [237,238]. The two trout alternatively spliced IFN-a transcripts translate into intracellular proteins that display similar antiviral functions to the secreted protein form but via interaction with cytosolic IFN receptors to activate the Jak/STAT signalling pathway. These data suggest that an active IFN system operates within virus-infected cells in fish.

The antiviral effect of the IFN-d subgroup is somewhat controversial. IFN-ds are the most common IFNs found in teleosts and exist as a single copy in cyprinids and salmonids but as multiple copies in the Acanthopterygiian species. Like the IFN-a group in cyprinids and salmonids, the IFN-ds are the major type I IFNs that activate the antiviral responses in Acanthopterygiians [214,219,239]. With the recent discovery of IFN-a in medaka, in addition to IFN-d, their antiviral activities have now been studied comparatively. Both IFNs increase expression of ISG15 and Gig genes in the medaka DIT cell line when overexpressed and can enhance cell resistance to viruses [219].

In contrast to the Acanthopterygiian IFN-d, the functions of IFN-d in cyprinids and salmonids are currently poorly studied. In Atlantic salmon, the FN-d gene is constitutively expressed in tissues but is not induced by viral mimics such as poly(I:C) or R848 [221]. However, in rainbow trout and zebrafish, it is activated during viral infection [207,208]. Salmon IFN-d has been produced as a recombinant protein in bacteria and HEK 293 cells but no activities have been detected [221].

The IFN-es are the latest member of the group I IFN family. Seven IFN-e genes have been found in rainbow trout [211] and are expected to be present in other salmonid species. Phylogenetically, trout IFN-es can be divided into two subgroups, one consisting of IFN-e1-4 and the other IFN-e5-7. They can be induced after stimulation with poly(I:C) and during viral infection but their functions remain to be characterised.

### 6.3. Group II IFNs

Group II IFNs have been identified in cyprinids, salmonids, and turbot, the latter belonging to the Pleuronectiforme family [207,209,210,220]. Akin to group I IFNs, group II IFNs also consist of three subgroups, IFN-b, -c and -f. The antiviral properties of IFN-bs and IFN-cs have been studied but not as extensively as group I IFNs, and no functional data are available for IFN-f. The IFN-b and IFN-c group are viral inducible cytokines and expression is largely restricted to certain leucocyte populations [210,221], suggesting different roles to the group I IFNs. Recombinant trout IFN-b1/IFN3 has been shown to weakly induce Mx expression in RTS-11 cells [210]. Later studies compared the antiviral activities of IFN-b and IFN-c in Atlantic salmon, indicating that both activate antiviral genes but that IFN-c is more potent than IFN-b [221]. Intramuscular injection with an IFN-c encoding expression plasmid gives strong protection of fish against ISAV challenge. In contrast, an IFN-b encoding plasmid provides much weaker protection [223]. In zebrafish, recombinant IFNφ2 (an IFN-c member) shows similar stimulatory effects to IFNφ1 (an IFN-a member) in terms of modulating viperin expression in cultured cells and they confer comparable resistance of fish larvae against Infectious Haematopoietic Necrosis Virus (IHNV) [207]. Interestingly, zebrafish IFNφ2 and IFNφ3 (both IFN-c members) and turbot IFN1 (a group II member) display weaker antibacterial effects and/or effects on pro-inflammatory gene expression than the group I IFNs [220,235].

Unlike mammalian type I IFNs, which bind to the same receptor complex consisting of IFNAR1 and IFNAR2, teleost group I and II IFNs activate different receptors to trigger cellular responses [207]. In zebrafish, group I (IFNφ1/4, IFN-a/d) and II (IFNφ2/3, IFN-c) IFNs share a common receptor chain (CRFB5) which is an orthologue of the mammalian IFNAR1 but each interacts with a ligand specific receptor; CRFB1 for group I and CRFB2 for group II IFNs [207]. The type I IFN receptors are even more complex in Atlantic salmon where eight copies of IFN receptor candidates have been found [240]. These include 3× CRFB5s, 2× CRFB1s, 1× CRFB2, 1× CRFB3 and 1× CRFBx. Consistent with studies in zebrafish, CRFB1a and CRFB2 mediate signal transduction of group I (IFN-a) and II (IFN-b/c) IFNs respectively. However, multiple CRFB5 paralogues seem to engage with different groups of IFN ligands. The IFN-a pairs with the CRFB5a, CRFB5b or CRFB5c, IFN-b with CRFBx and IFN-c with CRFB5a or CRFB5c respectively. In addition, intracellular forms of CRFB2 and CRFB5 exist in rainbow trout and are involved in activation of cellular responses by the intracellular IFN-a ligand [238].

The actions of type I IFNs are controlled by multiple transcription factors. Several studies have demonstrated that teleost fish type I IFNs signal via the conserved Jak-STAT pathway [203,205,206,241]. Many of these key transcription factors have now been characterised in fish. It has become apparent that zebrafish possess a complete set of Jak family members comprising Jak1, Jak2, Jak3 and Tyk2, with Jak2 further duplicated into two paralogues (Jak2a and Jak2b) [242,243]. Jak2a is suggested to be the functional orthologue of mammalian Jak2 [243]. The involvement of Tyk2 in IFN signalling has also been studied in Atlantic salmon. Structurally, salmon Tyk2 is organised into seven JAK-homology (JH) domains, with JH1 being the critical domain catalysing autophosphorylation [244]. In teleosts, multiple paralogues of STAT1 and STAT2 proteins (e.g., STAT1a, STAT2a and STAT2b) have been shown to control cellular signalling of type I IFNs [245].

The IFN responses can be negatively regulated by several transcription factors. The main negative regulators comprise the SOCS proteins and the protein inhibitor of activated STAT (PIAS) family. These transcription factors are highly conserved in sequence and protein structure and are expected to play similar roles to their mammalian counterparts in suppressing the IFN pathway in fish [246,247,248].

### 6.4. Type II IFNs

Type II IFN exerts regulatory roles in both innate and adaptive immunity, including activating macrophages, enhancing antigen presentation and promoting the Th1 T cell response. They are encoded by a single gene in mammals but in teleosts two members have been identified and are termed IFN-γ (IFN-γ2 in zebrafish and other cyprinids) or IFN-γ related molecule (IFN-γrel, or IFN-γ1 in zebrafish and other cyprinids) [249,250,251,252,253,254]. An IFN-γ orthologue is also present in the elephant shark [86].

In general, teleost IFN-γs display conserved functions with their mammalian orthologues (Table 1). The stimulatory effects of IFN-γ on fish monocytes/macrophages are well documented. IFN-γ is able to increase production of nitric oxide and reactive oxygen intermediates (ROI), and phagocyte activity in several species [254,255,256,257]. A recent report has demonstrated that carp IFN-γ2 (IFN-γ) promotes formation and activation of extracellular traps in macrophages and neutrophils to enhance their antimicrobial activity [256]. In IFN-γ treated cells, genes associated with the inflammatory response are markedly activated, highlighted by the elevated expression of IL-20L [258], IL-34 [60], TNF-α [58], CXCL11_L1 (γIP) [67,228,234,254], and iNOS [256]. However, IFN-γ has a weak stimulatory effect on the expression of IL-1β [20,161,255] and CXCL8 paralogues [67,255], synergising with IFN-γrel or LPS to increase their transcript levels [255,259]. Other genes shown to be induced by IFN-γ include the antiviral effector genes such as guanylate binding protein 1 (GBP1), Mx, and ISG15 [232,254], and those (e.g., gp91phox and p47phox) associated with NADPH oxidase activation [255].

Molecular evidence suggests that antigen presentation pathways are also modulated by IFN-γ. The transcripts of the MHC II gene are significantly increased in RTS-11 cells 24 h after treatment with IFN-γ [254]. In addition, global transcriptomic analysis has shown that several key genes involved in the MHC I antigen presentation pathway are also up-regulated by IFN-γ [57].

Cell-mediated T cell responses are also potentially modulated by IFN-γ in fish. Although Th cells have not been functionally characterised in fish, cytokines mainly involved in T cell immunity are now known to exist and have been shown to be altered by IFN-γ. For example, several γ-chain cytokines including IL-2 [56], IL-15 [161] and IL-21 [56], are induced by IFN-γ in trout or salmon leucocytes, confirming its role in promoting Th1 responses and memory T cell maturation. In contrast, the major cytokines (e.g., IL-17 isoforms) involved in Th17 responses are not affected [56].

Protective effects of IFN-γ against infection with intracellular pathogens have been examined in cultured cells and in fish. In trout RTS-11 cells, IFN-γ weakly induces Mx expression [254], whilst in salmon cells treated with IFN-γ, IPNV replication is inhibited and the CPE titres are substantially reduced. The protective effect is only partially dependent on IFN-α induction, since antibodies to IFN-a do not completely block the IFN-γ-mediated induction of Mx and ISG15 [232]. In addition, intraperitoneal injection of rIFN-γ activates immune responses against infection with *Edwardsiella tarda* in the olive flounder, resulting in significantly increased survival [260].

### 6.5. IFN-γrel

The IFN-γrel molecules share moderate homology with the teleost IFN-γs and are considered as a second member of the type II IFN family. The IFN-γrel proteins have recently been classified into two subgroups, namely IFN-γrel1 and IFN-γrel2, based on the presence of the nuclear localisation signal (NLS) at the C-terminus [261]. IFN-γrel1 contains a functional NLS whilst IFN-γrel2 does not. To date, most of the functional studies have been conducted in cyprinid species.

IFN-γrel is thought to be involved in immune defence against bacterial infection. The Rohu carp IFN-γrel gene is up-regulated after infection with *A. hydrophila*, *E. tarda* or *Shigella flexneri* [251]. The expression of IFN-γrel is also detected in LPS-stimulated common carp leucocytes enriched for B-cells [262]. In zebrafish embryos, knockdown of the IFN-γrel1 (IFN-γ1) and its cognate receptor CRFB13 by morpholinos results in a significant reduction of embryo survival after infection with the bacterial pathogen *Yersinia ruckeri*, suggesting it plays an important role in innate defence against bacterial infection [263]. When injected intramuscularly into Fugu, IFN-γrel increases expression of IL-1β, IL-6, p35, p40, and IFN-γ, which are important mediators of immune responses against bacterial infection [264]. In Fugu, IFN-γrel and IFN-γ appear to trigger similar responses, including phagocytic activity and lysozyme activity.

The activities of the IFN-γrel2 (IFN-γ1) protein have been studied extensively in goldfish monocytes/macrophages in parallel with IFN-γ [255]. Curiously, the goldfish IFNγrel2 is more potent than IFN-γ in inducing expression of iNOS, TNF-α2, CXCL8/IL-8 and ceruloplasmin, and in enhancing nitric oxide production and phagocytosis in monocytes/macrophages. Moreover, it has a stronger temporal priming effect on monocytes for ROI production but surprisingly down-regulates the priming effect of IFN-γ and TNF-α2. As shown in goldfish monocytes/macrophages, IFN-γrel signalling is mediated by the phosphorylation and nuclear transport of STAT1, a crucial transcription factor regulating IFN-γ signalling. However, in carp, no stimulatory effect of IFN-γrel on iNOS expression has been detected in phagocytes [259].

The antiviral effect of the two IFN-γrel subgroups have also been comparatively examined in the ginbuna crucian carp [252]. Both cytokines are able to protect fish against infection with crucian carp haematopoietic necrosis virus (CHNV), and monomers of both cytokines are active. Considering the fact that the IFN-γrel2 lacks the NLS motif, it has been suggested that the two IFN-γrels may exert overlapping antiviral functions but act in different compartments in fish cells.

### 6.6. IL-10

IL-10 is an anti-inflammatory cytokine and suppresses immune responses. The IL-10 gene has been identified in a number of teleost fish [200,265,266,267,268,269,270,271,272]. It exists as a single copy gene for most species but duplicated copies are present in rainbow trout. The herpesviruses of European eel and Koi carp also encode an IL-10 homologue [273,274,275].

The functions of IL-10 have been characterised in goldfish and carp [276] (Table 1). In general, the fish IL-10s, similar to the mammalian orthologues, act as a suppressor and exert a conserved role in dampening inflammatory responses. In goldfish monocytes activated with heat-killed *A. salmonicida*, incubation with IL-10 substantially decreases the expression of TNF-α1, TNF-α2, IL-1β1, IL-10, CXCL_8/IL-8, and the NADPH oxidase component p47(phox) [265]. On the other hand, pre-treatment of monocytes with 10–1000 ng/mL of IL-10 substantially abrogates *A. salmonicida* or IFN-γ activated ROI production. IL-10 also induces phosphorylation and nuclear translocation of STAT3 and rapid expression of SOCS3 [265]. Consistent with these observations, similar effects of carp IL-10 have been shown in PMA or LPS activated neutrophils and macrophages [276]. In addition, IL-10 inhibits expression of genes involved in MHC antigen presentation in carp macrophages.

Using an immunisation model, the roles of IL-10 have also been investigated in carp [276]. In HK cell cultures derived from fish immunised with *Trypanoplasma borreli* antigens, stimulation with IL-10 plus antigen significantly increases IgM antibody secretion. Interestingly, this stimulatory effect is not seen in PBL. When the HK cells from immunized fish were cultured with the *T. borreli* antigens alone or in combination with IL-10 for six days, the expression of cytokines and transcription factors associated with Th1 and Th2 responses was increased by antigen but decreased by the presence of IL-10, with a concomitant increase in CD8α2 and CD8β1 expression. These data suggest that carp IL-10 has differential effects on memory CD4+ and CD8+ T cells, and promotes B cell differentiation and IgM antibody secretion in an antigen specific manner.

A viral homologue of IL-10 has been identified recently in the Koi herpesvirus (Khv) genome and characterised functionally [273,274,275]. It has moderate homology with carp IL-10, and has a predicted signal peptide, but is not essential for viral replication *in vitro* or virulence *in vivo* [275]. In virus infected carp, the KhvIL-10 gene is highly expressed at the acute and reactivation phases. The KhvIL-10 increases the number of lyz+ cells when injected into zebrafish embryos, similar to the effect seen with zebrafish IL-10, and this activity can be inhibited by co-injection with IL-10R1 morpholinos [274]. A recent study has extended the functional analysis where the KhvIL-10 is shown to exert overlapping functions with the host IL-10, as an anti-inflammatory factor via the STAT3 signalling pathway [273]. These data demonstrate that fish herpesviruses produce a functional IL-10 analogue to suppress the host immune response to establish infections.

### 6.7. Other Members of the Type II α-Helical Cytokine Family

Other members of the type II α-helical cytokine family include IL-19, IL-20, IL-22, IL-24 and IL-26. The genes encoding IL-19, IL-20 and IL-24 are clustered with the IL-10 gene, whilst the IL-22 and IL-26 genes reside next to IFN-γ at a separate locus. In teleosts, a cytokine homologue related to the mammalian IL-19/IL-20/IL-24 genes, termed IL-20 like (IL-20L) in trout, has been identified [200,258]. Teleost fish also possess orthologues of IL-22 and IL-26 [242,250,277,278]. Among these cytokines, only the activity of the trout and So-iny mullet IL-22 has been described to date [277,278]. In trout splenocytes, IL-22 can up-regulate the expression of AMP genes including β-defensins 3 and 4, liver expressed antimicrobial peptide 2A and hepcidin [277] (Table 1). Furthermore, administration of the IL-22 into mullet significantly improves fish survival after challenge with the Gram positive bacterial pathogen *Streptococcus dysgalactiae* [278]. Like mammalian IL-22, fish IL-22s are expected to play a major role in co-ordinating immune defence against bacterial pathogens, and their expression has been shown to correlate with vaccine-induced protection [279].

## 7. Conclusions

There is a clear conservation of the cytokine network throughout vertebrates, with many of the key cytokines important for haematopoiesis, inflammation and adaptive immunity present from fish to mammals. However, in many cases, the genes are not true homologues and may be related to ancestral genes that have diverged independently in different vertebrate lineages. Hence the importance of determining the protein function in each species studied. Nevertheless, the data obtained to date, and reviewed here, show that so far there have been relatively few surprises. Fish cytokines have a role in development and haematopoiesis, in line with knowledge from mammalian studies. They attract leucocytes to a site of infection and activate their antimicrobial mechanisms to kill any invaders. The cytokines present have the potential to influence the type of response elicited, with classical Type 1 and Type 2 immunity likely controlled by the release of appropriate cytokines from innate lymphoid cells or helper T cells. This information gives us an insight into what may have been the minimal cytokine network needed for an early adaptive immune system, to allow appropriate expansion of antigen-selected cells without the risk of excessive host damage. In addition, as we characterise the cell populations that release the different cytokines present, we will begin to realise the potential for manipulating such responses, with many possible applications. For example, they may be useful as markers of effective vaccination of fish in aquaculture, or for selective breeding of “robust” fish to improve fish welfare. The diversity of fish species can be a confounding factor in our efforts to characterise these genes and the functions of the encoded proteins but it is clear that a few key fish species are driving the field forwards, from economic necessity or the ability to perform genetic manipulations *in vivo*. So indeed it is true to say that we do now know a lot about the function of many fish cytokines, and this is aiding our ability to characterise the fish immune system and pathways leading to protective immunity.

## Figures and Tables

**Table 1:** Biological activity of fish type II cytokines.

Cytokine	Human homologue	Receptors	Known immune functions	references
IL-10, viral IL-10	IL-10	IL-10R1/CRFB12, IL-10R2/CRFB4	Suppress immune responses, inhibit inflammation, promote T cell proliferation, memory B cells, and IgM production	[265,273,276]
IL-20L	IL19, IL-20, IL-24	Not characterised	Not characterised	[200,258]
IL-22	IL-22	Not characterised	Activate antimicrobial peptide genes and antibacterial immunity	[277,278]
IL-26	IL-26	Not characterised	Not characterised	[242,250]
Group I IFN (IFN-a, d, e)	Type I IFN	IFNAR1/CRFB5, IFNAR2/CRFB1	Induce expression of the antiviral effectors, promote apoptosis, regulate inflammation	[201,207,210,221,234]
Group II IFN (IFN-b, c, f)	Type I IFN	IFNAR1/CRFB5, IFNAR2/CRFB2	Induce expression of the antiviral effectors, promote apoptosis	[207,210,221,223]
IFN-γ	Type II IFN	IFN-γR1/CRFB13, IFN-γR2/CRFB6	Activate phagocytes, enhance antigen presentation, promote Th1 cytokine expression	[254,255,256,257]
IFN-γrel	Type II IFN	CRFB17, IFN-γR2/CRFB6	Regulate anti-bacterial and antiviral immunity	[255,261]

## Abbreviations

The following abbreviations are used in this manuscript:

CCK	CC chemokine
CD	cluster of differentiation
CISH	cytokine inducible Src homology 2 (SH2)-containing protein
CNTF	ciliary neurotrophic factor
COX-2	cyclooxygenase-2
CRFB	class II cytokine receptor family member
CSF	colony stimulating factor
DAMP	danger associated molecular pattern
DC-SIGN	dendritic cell-specific intercellular adhesion molecule-3-grabbing non-integrin
DIGIRR	double immunoglobulin IL-1R related molecule
γIP	gamma interferon inducible protein
GBP	guanylate binding protein
G-CSF	granulocyte colony stimulating factor
GM-CSF	granulocyte-macrophage colony-stimulating factor
HK	head kidney
HSC	haematopoietic stem cell
IGFBP	insulin growth factor binding protein
ICE	IL-1β converting enzyme
IEC	intestinal epithelial cell
IFN	interferon
IFN-γrel	interferon gamma related molecule
Ig	immunoglobulin
IL	interleukin
IL-1F	interleukin-1 family member
IL-1Ra	interleukin 1 receptor antagonist
IL-1RAcP	interleukin-1 receptor accessory protein
IL-36Ra	interleukin-36 receptor antagonist
ILC	innate lymphoid cell
iNOS	inducible nitric oxide synthase
IRAK	interleukin 1 receptor associated kinase
IRF	interferon regulatory factor
ISG	interferon stimulated gene
JAK	Janus kinase
LPS	lipopolysaccharide
LT	lymphotoxin
MAPK	mitogen-activated protein kinase
M-CSFR	macrophage colony stimulating factor receptor
MyD88	myeloid differentiation factor 88
NF-κB	nuclear factor kappa-light-chain-enhancer of activated B cells
nIL-1Fm	novel interleukin-1 family member
PBL	peripheral blood leucocyte
PAMP	pathogen associated molecular pattern
poly(I:C)	Polyinosinic-polycytidylic acid
PRR	pattern recognition receptor
ROS	reactive oxidative species
SIGIRR	single domain interleukin-1 receptor related molecule
SOCS	suppressors of cytokine signalling
STAT	signal transducers and activators of transcription
TACE	TNF-α-converting enzyme
TGF	transforming growth factor
TIR	Toll/IL-1 receptor
TNF	tumour necrosis factor
TNFSF	tumour necrosis factor superfamily member
TNFSFR	tumour necrosis factor superfamily member receptor
TRAF	tumour necrosis factor receptor associated factor
Treg	regulatory T cell
Tyk	tyrosine kinase
WGD	whole genome duplication
